# The vitamin D paradox in Black Americans: a systems-based approach to investigating clinical practice, research, and public health - expert panel meeting report

**DOI:** 10.1186/s12919-018-0102-4

**Published:** 2018-05-09

**Authors:** LaVerne L. Brown, Barbara Cohen, Derrick Tabor, Giovanna Zappalà, Padma Maruvada, Paul M. Coates

**Affiliations:** 1 Office of Dietary Supplements, National Institutes of Health, Bethesda, MD USA; 2 National Institute of Minority Health and Health Disparities, National Institutes of Health, Bethesda, MD USA; 3 National Institute on Aging, National Institutes of Health, Bethesda, MD USA; 4 National Institute of Diabetes and Digestive Kidney Diseases, National Institutes of Health, Bethesda, MD USA

**Keywords:** Vitamin D, Bone health, Dietary reference intakes, Black vs. White Americans, Paradox

## Abstract

The Office of Dietary Supplements, the National Institute on Minority Health and Health Disparities, the National Institute on Aging, and the National Institute of Diabetes and Digestive and Kidney Diseases, all components of the U.S. National Institutes of Health, co-sponsored an expert panel meeting to discuss the vitamin D paradox in Black Americans. The paradox is that despite markedly low (or “deficient”) measures of vitamin D status in Black Americans, the incidence of falls, fractures, or osteopenia are significantly lower compared to White American counterparts with similar vitamin D status. Six panelists were invited to engage in guided discussions on the state of the science with respect to key knowledge gaps impacting vitamin D status and bone health. They were also asked to reflect on best approaches for advancing the science.

A central theme throughout the discussions was that there may be many factors that impact Vitamin D levels in Black Americans and understanding these factors may be key to understanding mechanisms for improving bone health in all populations. Data presented showed that although adiposity, skin pigmentation, vitamin D binding protein polymorphisms, and genetics all contributed to differences in 25(OH)D levels in Black vs. White Americans, no one factor alone could fully explain the vitamin D paradox in Black Americans. However, the panelists did agree that the paradox is significant and warrants further investigation. There was consensus that Black Americans gained no skeletal benefits from high doses of vitamin D supplementation, and that high levels of the biomarker of vitamin D status, serum 25-hydroxyvitamin D or 25(OH)D, in this population are almost certain to result in adverse effects. Some panelists proposed that additional studies are needed so that the Institute of Medicine (IOM) can better define the safe upper limits of vitamin D intake in this and other subpopulations. Others suggested a need for better, more generalizable biomarkers of bone health to advance the science.

## Background

Cholecalciferol, or vitamin D, is a prohormone that is synthesized endogenously in mammalian skin upon exposure to sunlight or UVB irradiation (290-315 nm). A steady state is reached such that only 10-15% of the cutaneous precursor, 7-dehydrocholesterol, is converted to vitamin D [1]. Such photo-regulation is believed essential to prevent the production of toxic levels of vitamin D following excessive sun exposure [1]. However, additional sources of vitamin D can be obtained via the diet in small amounts from foods such as salmon, tuna, mackerel, beef liver, and egg yolks, and in larger amounts from fortified foods or dietary supplements. Regardless of the source, vitamin D must be further metabolized or “activated” before exerting pleiotropic actions at various tissue and cell sites throughout the body.

The first step in the activation of cholecalciferol takes place in the liver. There, vitamin D is converted to 25-hydroxyvitamin D (referred to as 25(OH)D). The 25(OH)D metabolite is most abundant in the circulation, and it is an established marker of vitamin D status. The 25(OH)D metabolite is, however, inactive at physiological levels [1]. The second step in the activation of vitamin D takes place in the kidneys and other organs/tissues where 25(OH)D is converted to 1,25(OH)_2_D. Formation and catabolism of this, the active metabolite (1,25(OH)_2_D), are tightly regulated processes such that when circulating levels of 25(OH)D are low, levels of 1,25(OH)_2_D have been shown to be normal or somewhat elevated.

The biological actions of 1,25(OH)_2_D are mediated through the vitamin D receptors (VDRs), which are found at multiple cell and tissue sites. The VDRs are most abundant in the intestine, kidney, parathyroid gland, and bone, and their expression at these sites is linked to calcium homeostasis [2]. As such, vitamin D and its active metabolite(s) are conventionally associated with health outcomes related to bone strength and condition.

### The vitamin D paradox

The committee assigned by the IOM to review dietary reference intakes (DRIs) for vitamin D and calcium determined that, with respect to the prevention of fractures and osteopenia, levels of serum 25(OH)D were sufficient when in the range of 20 ng/mL- 50 ng/mL; low when less than 20 ng/mL; and deficient when below 12 ng/mL [3]. In addition, assuming minimal sun exposure, an intake of 400-600 IU per day was deemed adequate to maintain serum 25(OH)D levels in the range of 16-20 ng/mL [3]. While studies show a correlation of serum 25(OH)D levels to bone mineral density and fracture risk in White and Mexican-Americans, serum 25(OH)D levels do not correlate with the same health outcomes in some other populations, particularly Black Americans. In Black Americans, bone density levels are high despite markedly low or deficient serum levels of 25(OH)D. The apparent contradiction is commonly referred to as the “vitamin D paradox in Black Americans”.

A quote from the National Academies Press in 2011 reads, “…emerging evidence would suggest that there is perhaps a lower requirement for calcium and vitamin D among African Americans relative to ensuring bone health, at least compared with whites, [but] there is a notable lack of high-quality and convincing evidence to act on this possibility…” [3]. In 2014, an ODS-sponsored workshop, “Vitamin D: Moving Toward Evidence-based Decision Making in Primary Care,” identified additional ambiguities regarding the efficacy and potential risks of vitamin D supplementation with respect to skeletal health amongst Black Americans [4].

## Overview of presentations

To continue discussions on the state of the science with respect to vitamin D requirements in specific segments of the population, and to gain a better understanding of the factors capable of affecting those requirements, the ODS, the NIMHD, the NIA, and the NIDDK co-sponsored an expert panel meeting in December 2017 entitled, “The Vitamin D Paradox in Black Americans: A Systems-based Approach to Investigating Clinical Practice, Research, and Public Health.” The systems-based approach was defined as the study of a series of factors that impact a whole system by way of individual actions, as well as through interrelated and interconnected interactions with other components. A primary aim of the expert panel meeting was to consider the influence of interrelated factors that might impact the vitamin D status and bone health in Black vs. White Americans. Some plausible factors include: behavioral (e.g., physical activity, diet, sun exposure, etc.), molecular (e.g., calcium, parathyroid hormone or PTH, etc.), clinical (e.g., obesity vs. weight loss), or physiological (e.g., skin pigmentation, genetics). Social factors (e.g., access to quality healthcare) were deemed to be outside the scope of this forum.

Six panelists, each with professional expertise in the realm of the vitamin D paradox, were invited to the meeting. The panelists were invited to engage in informal discussions addressing specific themes of the vitamin D paradox to gain a better understanding of the factors impacting vitamin D status and bone health in various subpopulations in the U.S. Prior to the discussions, panelists presented clinical data or research findings that best supported their expert opinions of the vitamin D paradox. The charge was to focus each presentation on the impact of 25(OH)D as a marker of skeletal health in isolation but also in connection with other factors (e.g. calcium status, adiposity, genetics, skin pigmentation, etc.) in Black American vs. White American adult populations. Panelist presentations began with an overview of vitamin D pathways followed by an overview of the Vitamin D Standardization Program (VDSP). The VDSP has served an important role in helping to advance the science and improve the clinical assessment of nutrient status in general. The following summarizes all presentations as well as both sessions of the expert panel discussions on the vitamin D paradox.

### Panelist: Andy Hoofnagle, MD, PhD, Department of Laboratory Medicine, University of Washington

**Fig. 1:**
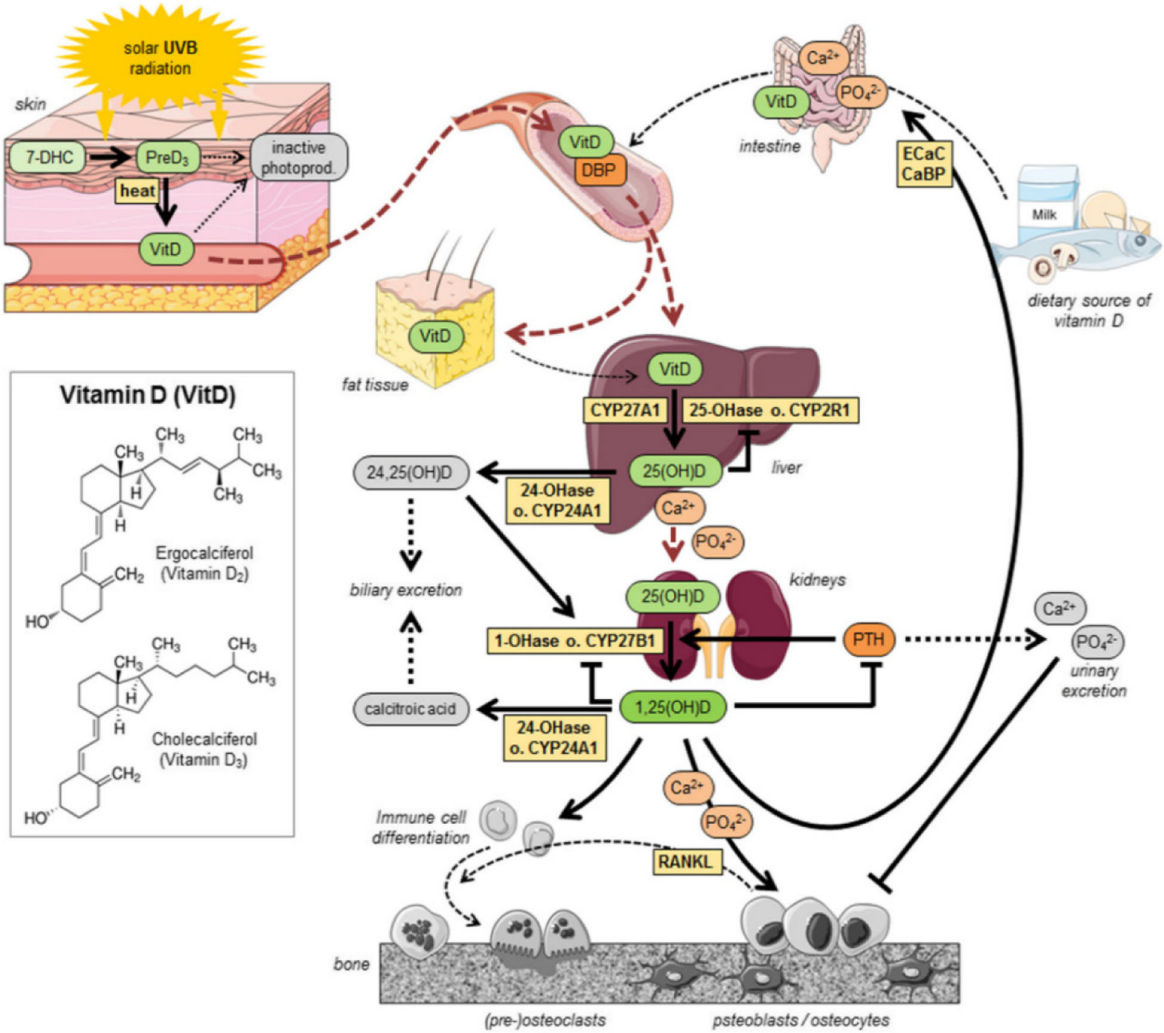
Vitamin D Pathways: An Overview (source: *Nutrients* 2016, *8*, 319) [6]. The conversion of vitamin D to the active metabolite, 1,25(OH)_2_D, is shown. The pathway starts with the exposure of vitamin D from dietary sources or via cutaneous synthesis following UVB radiation. Vitamin D is bound to VDBP and transported to the liver where it is converted to 25(OH)D, the current biomarker for vitamin D status. PTH enhances 1-alpha hydroxylase in the kidneys which is responsible for the conversion of the inactive metabolite, 25(OH)D, to the active metabolite, 1,25(OH)_2_D. The entire pathway is tightly regulated via catabolic and feedback loop processes as shown

Dr. Hoofnagle presented a review of some fundamentals of the vitamin D pathways (Fig. 1) that may be relevant in addressing a key question: How can biochemical information be used to help predict clinical outcomes? The role of PTH in enhancing the activity of the enzyme 1-alpha hydroxylase in the conversion of 25(OH)D to 1,25(OH)_2_D was discussed as well as the role of PTH in inhibiting 24 hydroxylase.

Dr. Hoofnagle also presented data from the multi-ethnic study of atherosclerosis (MESA). The multi-site study enrolled 6814 participants from North Carolina, New York, Maryland, Minnesota, Illinois, and California. The participants were 45-84 years of age, 53% female, and of African American, Chinese American, White, and Hispanic ethnicities. After ≥8 years of follow-up, key markers of the vitamin D pathway were monitored. Vitamin D binding globulin, albumin, and fibroblast growth factor 23 or FGF23 showed no significant differences between races (Black vs. White Americans). Measures of calcium and phosphate also showed no difference, but PTH was higher in Black Americans than in White Americans. Consequently, it was not surprising that compared to White Americans, Black Americans displayed lower levels of 25(OH)D, lower levels of the catabolite 24,25(OH)_2_D, and higher levels of the active metabolite 1,25 (OH)_2_D. In addition, 25(OH)D levels appeared to decrease as skin color darkened when comparing White, Chinese, Hispanic, and Black Americans.

With respect to health outcomes, the MESA trial showed that higher serum levels of 25(OH)D were associated with lower cardiovascular (CVD) risk in White and Chinese Americans, while higher serum levels of 25(OH)D were associated with a slightly higher risk of CVD in Black and Hispanic Americans. In addition, the fracture rate for Black Americans was lower than that for White Americans.

In a related study using the National Health and Nutrition Examination Survey (NHANES) data from 1988 to 2010 [using national death index, ages 20+, analytic sample *N* = 39,839], the all-cause mortality rate was increased for White Americans when compared to Black Americans, and higher levels of 25(OH)D were associated with lower all-cause mortality in White Americans but less strongly associated with all-cause mortality in Black Americans.

### Presenter: Chris Sempos, Ph.D. NIH Office of Dietary Supplements

Dr. Sempos’ presentation focused on assay variation and end-user performance that tend to bias measurements of vitamin D status. The identification of inadequate and/or deficient vitamin D levels, and related health consequences in individuals and populations, requires laboratory measurements that are accurate, precise, and comparable over time, location and laboratory procedure. The same level of standardization is required to identify the health effects of vitamin D overload.

Results from the Vitamin D External Quality Assessment Scheme (DEQAS) and research around the world clearly indicate that assay variation in the measurement of 25(OH)D, the current biomarker of vitamin D status and exposure, confounds our ability to develop external criteria to define inadequate and deficient levels of 25(OH)D. Thus, current laboratory procedures require standardization. With the development of standard reference measurement procedures for 25(OH)D, first by the US National Institute of Standards and Technology, and then by the Laboratory for Analytical Chemistry at Ghent University in Belgium, it is now possible to contemplate the worldwide standardization of 25(OH)D measurements.

The path to 25(OH)D assay standardization includes four stages:

Step 1. Develop a Reference Measurement System. Such a system consists of reference methods, reference materials, a certification program and Accuracy-based Performance Testing/External Quality Assessment (PT/EQA) schemes.
Step 2. Calibrate Commercial Assay Systems to Reference Materials. Examples of this include the National Institute of Standards and Technology’s (NIST) Standard Reference Materials (SRMs) or the CDC Vitamin D Standardization Certification Program that are based on single donor serum panels with target values assigned by a reference measurement procedure.
Step 3. Calibrate Individual Clinical and Research Laboratory Assays to Reference Methods (Materials). This includes NIST SRMs, CDCs Standardization Certification Program and participation in CAP and/or DEQAS PT/EQA.
Step 4. Verify End-User Test Performance. The CDC Standardization Certification Program, as well as the CAP Accuracy-Based Vitamin D Survey and DEQAS are useful ways to continuously verify end-user performance over time.

**Fig. 2:**
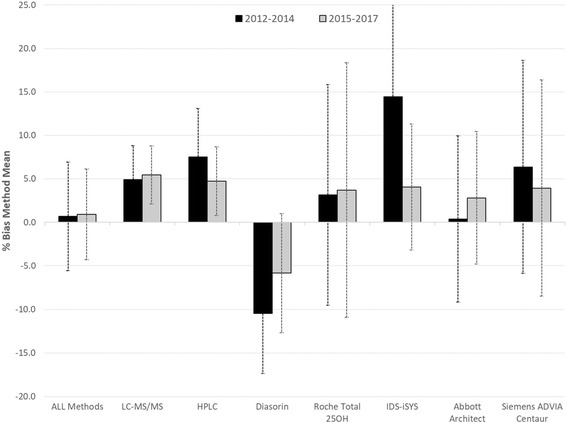
Comparison of percent bias of 25(OH)D Assays [7]. Comparison of % bias of Method Mean of 25(OH)D assays from NIST-assigned target values for DEQAS samples from 2012 to 2014 and 2015-2017. The error bars are ± SD of the % bias (*n* = 36). Adapted from C.Q. Burdette et al., J. AOAC Int., 100 (5), 1277-1287 (2017)

A comparison of different assays shows a different bias between 2012 and 14 and 2015-17 (see Fig. 2). There is still a large amount of variation around mean bias for most assays – especially the immunoassays. These results highlight the need to modify the VDSP Performance Criteria for Mean Bias to include an allowable amount of variation around a mean of ±5%.

The problem remains -- how these data, with such variability, can be pooled. In addition, four key emerging issues for vitamin D research include:

1. Developing updated VDSP Performance Guidelines for the measurement of 25(OH)D.
2. Emerging importance of additional vitamin D metabolites in assessment of vitamin D status.
3. Developing standardization programs for emerging vitamin D metabolites.
4. Promoting consensus definition of vitamin D deficiency based on standardized measurements of 25(OH)D.

Dr. Sempos was clear that the current goal is to promote the standardized laboratory measurement of vitamin D status to improve clinical and public health practice worldwide and to define vitamin D status. One potential definition focuses on specific concentrations of one of the following: 25(OH)D; 24,25(OH)_2_D_3;_ 3-epi-25(OH)D; PTH Suppression by 25(OH)D; vitamin D binding protein; or, bioavailable 25(OH)D or something similar. Another definition considers a combination of vitamin D metabolites.

The expansion of VDSP vitamin D standardization efforts (through ODS) includes the promotion of standardized measurement of emerging vitamin D metabolites in research; the development of NIST reference methods or reference materials (e.g., 3-epi-25(OH)D and 24,25(OH)_2_D); and, the continued development of NIST reference methods for vitamin D binding protein (DBP) and PTH.

Given that, when trying to improve the definition of vitamin D deficiency most researchers use the relationship between 25(OH)D and the risk of developing nutritional rickets, future goals could include the development of: an international rickets registry; a case definition of nutritional rickets; standardized 25(OH)D/Vitamin D metabolites; standardized nutritional rickets risk factors (dietary calcium intake, iron status, etc.); and, a consensus on hypovitaminosis D definition.

### Panelist: John Aloia, MD, Stony Brook University School of Medicine

**Fig. 3:**
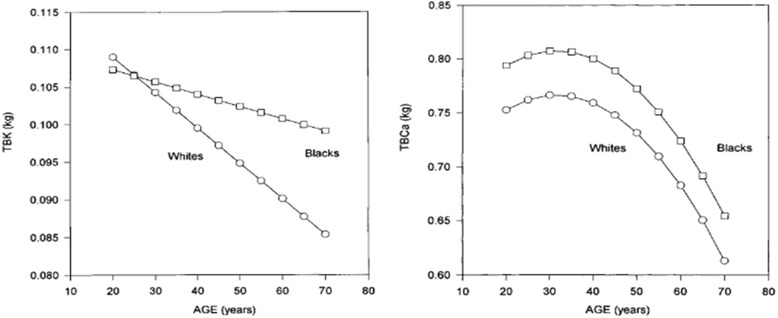
Differences in skeletal and muscle mass with aging in Black and White women (source: *Am J Physiol Endocrinol Metab,* 2000*, 278*(6), E1153-1157) [8]. Change with age in total body potassium (TBK) in black and white women, adjusted to mean height and weight for each race. For blacks, TBK = 0.11057 − 0.00016409 × Age (*P* < 0.0424). For whites, TBK = 0.11841 − 0.00047214 × Age (*P* < 0.0001). b) Total body calcium (TBCa) plotted against age in black and white women by use of a quadratic model, adjusted to mean height for each race. For blacks, TBCa = 0.70415 + 0.0065622 × Age − 0.00010433 × Age2 (*P* < 0.0001). For whites, TBCa = 0.66308 + 0.0065622 × Age − 0.00010433 × Age2 (*P* < 0.0001)

Citing work that he has been involved with since the early 1970’s, Dr. Aloia stated that Black Americans have higher bone mass when compared to White Americans. He presented data that show clear differences in total body potassium and calcium in White and Black Americans (Fig. 3), along with data that show that Black Americans have higher mineral and protein mass than White Americans (Table 1).

**Table 1:** Body composition variables for Black and White Americans [9]

Variable	Blacks % (SE)	Whites % (SE)	B/W	*P*-value ^a^
Mineral	4.37 (.052)	4.10 (.037)	1.066	.0001
Fat	34.1 (.97)	35.6 (.63)	0.958	.054
Protein	13.9 (.097)	13.4 (.078)	1.037	.0001
Water	47.6 (.37)	46.9 (.27)	1.015	.025

Values given are at Age 47.7, Height 163.6 cm, Weight 66.9 kg. Values are percent of total body weight, obtained by using separate equations for the weight of each compartment and then computing the percentage of the total compartments

^a^ The *P*-value given is the result of Student’s *t*-test on the difference between the percentages

In looking at non-vitamin D factors that impact bone health, Dr. Aloia indicated that Black Americans displayed genetically programmed bone mass, higher peak bone mass, greater muscle mass, advantageous femur geometry, superior calcium economy, skeletal resistance to PTH, and renal calcium conservation. He also believed that 60 – 80% of peak bone mass could be attributed to genetic inheritance since differences were observed in all age groups, not just in older populations.

With respect to calcium economy in Black adults, Dr. Aloia presented findings showing that Black Americans have: reduced markers of bone turnover (osteocalcin, CTx, OHPro, BAP); lower urinary calcium; similar calcium absorption efficiency; higher PTH and 1,25(OH)_2_D; skeletal resistance to PTH; and, slower bone loss when compared to White Americans. Ultimately, Dr. Aloia suggested that, given such lower concentrations of urinary calcium in Black individuals, the IOM should consider lowering the ranges in the recommended calcium guidelines for Americans.

With respect to the use of free 25(OH)D versus total 25(OH)D assays for assessing vitamin D status, Dr. Aloia indicated that a study with 164 Black and White Americans matched for age and BMI showed higher PTH and 1,25(OH)D levels in Black Americans. There was no advantage of using free 25(OH)D versus total 25(OH)D for predicting outcomes, and the VDBP levels were the same in both groups.

Dr. Aloia highlighted his findings from a 3-year, randomized, double-blind, placebo controlled, parallel-group study to evaluate the efficacy of vitamin D3 in the prevention of bone loss in 208 black postmenopausal women. The study results showed no change in bone mineral density (BMD) with vitamin D supplementation (i.e., 800 International Unit or IU for the first 2 years and 2000 IU for the last year of the trial) [5].

Dr. Aloia concluded his remarks with the following statement: “A dilemma that exists is that higher levels of vitamin D presents hazards for Black Americans, resulting in increasing falls and fractures. Yet Black Americans with low levels (less than 30 nmol/l) are at greater risk for osteomalacia or rickets. So, if we lower the levels we run the risk of causing more osteomalacia and rickets.”

### Panelist: Steve Brooks, PhD, Health Canada

Dr. Steve Brooks presented data from both the Canadian Health Measures Survey and a survey of South Asian and White Adults in Ottawa. Both surveys used self-reported ethnicity, food frequency questionnaires for vitamin D from food and supplements, and clinical measures that included serum 25(OH)D and natural skin color by spectrophotometer (unexposed - upper arm underneath biceps).

The data showed lower levels of 25(OH)D in Black Canadians compared to White Canadians and a large proportion of non-White Canadians with levels below 20 nmol. He noted that levels in Black Canadians were even lower than levels in Black Americans.

Based on the survey data, three key questions need to be addressed.

1. Do we need to worry about lower 25(OH)D levels in “non-White” groups?
2. Are the Dietary Reference Intakes (DRI) values (derived for optimal bone health) correct for all racial groups?
3. Are there optimal “race-specific” values? (e.g., considering data on Black Americans with respect to VDBP and osteoporosis)?

Dr. Brooks indicated that the usual explanation for low levels of serum 25(OH)D in non-White populations was skin color. He alluded to data that suggested that during the summer months, darker people do not synthesize as much vitamin D as people with lighter skin, signifying that the rate of synthesis is lower with darker skin.

The Ottawa study measured skin color and serum levels of 25(OH)D. It was designed to analyze the impact of skin color separately from the impact of other genetic differences. Results from the study showed that South Asians have darker skins than whites and have a wide range of color. There is a slight decrease in vitamin D among South Asians, as skin color gets darker. However, the difference between South Asians and Whites was remarkable (25 nmol/L). Thus, the data suggest that the differences in 25(OH)D levels could be due to other unexplained factors, with genetics being one of them. Also, sunbathing/tanning was believed to influence the results in the White group. Comparisons of the differences in 25(OH)D levels during the fall and spring months of South Asians versus White Canadians, with respect to sex, age, BMI, cholesterol and vitamin D intake (food and supplement), led Dr. Brooks to believe that these different 25(OH)D levels are at least partially explained by race.

Dr. Brooks concluded his remarks with three summary statements:

- While there is a skin color effect, we don’t understand the practical meaning of this effect.
- Skin color is not the only reason for the differences in 25(OH)D levels among the racial/ethnic groups -- there could be a genetic determinant of 25(OH)D levels in different racial/ethnic groups.
- While we can account for intake (Food Frequency Questionnaire or FFQ, supplement) and exposure (UVB ground level, time outside), none of these measures are perfect and an unaccounted for factor or interaction may exist.

### Panelist: Keith Norris, MD, PhD, UCLA Division of General Internal Medicine and Health Services Research

Dr. Keith Norris began his presentation with the following clinical observations:

- Compared to White dialysis patients, Black dialysis patients have increased intact-parathyroid hormone or iPTH levels and yet despite these higher levels, Black dialysis patients have increased bone mineral density and lower fracture rates.
- There is a greater likelihood of survival in Black dialysis patients who received activated vitamin D versus no/low dose vitamin D, but a similar increased survival is not seen in White dialysis patients.
- There were no Black-White mortality differences in dialysis patients in upper 50th percentile of FGF23 levels, but there was a 60% lower mortality for Blacks if FGF23 levels in the lower 50th percentile.

He urged the panelists to consider a balance between bone and other outcomes associated with vitamin D.

Dr. Norris also presented data that considered serum PTH as a function of 25(OH)D. The data showed that PTH was not linear in Black participants and that multivariable-adjusted change in PTH (95% CI) per 1 ng/mL change in 25(OH)D above and below 20 ng/mL was significant for White & Hispanic, but not Black participants. Furthermore, changes in iPTH for varying 25(OH)D levels were muted in Blacks or there may be more changes in PTH fragments or oxidized PTH such that iPTH does not need to change much.

In addition, Dr. Norris presented data on mean PTH concentrations, dietary calcium intake, and whole body bone mineral density (BMD) in NHANES 2003–2004, stratified by race/ethnicity and by categories of 25(OH)D (ng/mL). He indicated that lower serum 25(OH)D levels were associated with lower BMD in White and Hispanic participants, but there was no change in BMD in Black participants. He concluded that compared to other racial or ethnic groups Blacks do not have a significant relationship between 25(OH)D levels and PTH or BMD.

Dr. Norris then focused on non-bone markers associated with vitamin D status. He noted a study similar to MESA study that examined racial differences in the association of serum 25(OH)D concentration with congenital heart defect (CHD) events. The study showed a strong relationship between 25(OH)D and CVD events in Whites and Chinese, but not in Blacks or Hispanics.

On the topic of whether there might be better markers of vitamin D status and bone health, Dr. Norris considered the 24,25(OH)_2_D catabolite. He noted that while both 25(OH)D levels and 24,25(OH)_2_D levels were higher in White Americans compared to Black Americans, the ratio of 25(OH)D to 24,25(OH)_2_D was the same in both groups.

Dr. Norris concluded his presentation with the following summary statements:

- Mean PTH in Black Americans with low turnover bone disease is equivalent to mean PTH in Whites with high turnover bone disease.
- Racial differences in PTH may be due to differences in the ratio of intact PTH to PTH fragments (possible blocking fragments); racial differences in oxidized and non-oxidized PTH (both recognized as iPTH but oxidized PTH is inactive); or, limitations of PTH assays.
- Based on studies examining serum 25(OH)D levels, racial differences exist in the vitamin D - PTH axis and its relationship to multiple bone and mineral conditions as well as immune and cardiac related pathways.
- Present measurements of iPTH may not capture fragments or oxidation that influence active PTH and may vary by race.
- Changes in serum 25(OH)D have commensurate changes in serum 24,25(OH)_2_D and the 24,25(OH)_2_D/25(OH)D ratio may be more specific and may be less sensitive to differences by race.
- The 24,25(OH)_2_D/25(OH)D ratio may reflect free 25(OH)D and/or differences in VDBP levels and/or polymorphisms.

### Panelist: Camille Powe, MD, Massachusetts General Hospital

Dr. Camille Powe’s presentation was focused on a genome wide association study (GWAS) of 25(OH)D concentrations in 33,996 individuals of European descent from 15 cohorts. Of the 33,996 individuals, 16,125 subjects were amongst the five cohorts that were designated as discovery cohorts. Results from the discovery cohort showed three genetic variants (or hits) associated with areas of the genome that were linked to vitamin D levels. The hits were confirmed in replication cohorts. The top hit was the GC (GC-globulin or group-specific component, vitamin D binding protein or VDBP) while the other two were: 7-dehydrocholesterol reductase or DHCR7 (converts 7DHC to cholesterol); and, cytochrome P450 family 2 subfamily R member 1 or CYP2R1 (involved with 25-hydroxylation of vitamin D in the liver). Dr. Powe focused her discussions on GC and VDBP variant types.

Dr. Powe considered whether racial differences in VDBP might explain the paradox. She explained that vitamin D metabolites are hydrophobic and circulate via carrier proteins such as VDBP. She also stated that since the free hormone hypothesis states that only unbound proteins have biological actions, she and her colleagues considered the VDBP in the context of health outcomes associated with vitamin D. Dr. Powe presented studies that showed that free vitamin D levels in Blacks and Whites were similar. Since the affinity for 25(OH)D differed with respect to VDBP variant type, it seemed possible that the VDBP variant type might influence the amount of vitamin D that is free.

Two polymorphisms in the VDBP gene were identified: rs7041 and rs4588. These coding single nucleotide polymorphisms appeared to result in amino acid changes in the protein that were linked to changes in Gc1 s, Gc1F and Gc2. Most self-identified Blacks in the GWAS were 93% homozygous for Gc1f, while among white homozygotes most (75%) had the Gc1 s variant. There was a minority with the Gc2 variant.

Dr. Powe presented the following summary statements.

- GWAS identified genetic variants associated with total 25(OH)D levels that include common variants in the VDBP gene and other variants in loci potentially related to vitamin D metabolism (DHCR7, CYP2R1).
- Coding variants rs7041 and rs4588 in the VDBP gene track with ancestry (recent African vs. European); result in amino acid changes in the VDBP protein; and, associate with total 25-hydroxyvitamin D levels.
- Quantitative and qualitative differences in VDBP impact vitamin D biology. In vitro, VDBP variants differ in their affinity for 25(OH)D, while in mice models fed a standard diet, low VDBP levels result in sufficiency (preserved calcium balance and adequate intracellular concentrations of 1,25(OH)_2_D) at lower total 25(OH)D levels, and in humans, VDBP variants affect 25(OH)D half-life.

In conclusion, Dr. Powe believed that VDBP genetics might contribute to the vitamin D paradox in Black Americans. She cautioned that researchers should be careful not to combine race and ancestry. She emphasized that while genetic ancestry is associated with race, people self-identifying as black actually have various genetic ancestries. In fact, there is a large range of ancestries among people of similar self-identified races.

### Panelist: Sue Shapses, PhD, RD, Department of Nutritional Sciences, Rutgers University

Dr. Sue Shapses began her presentation with an overview of how special populations were handled in the IOM report for vitamin D guidelines. She indicated that the IOM report was focused on the general population, and the guideline recommendations were intended for at-risk individuals. However, the guidelines did incorporate some special conditions that were a concern for the general population. The conditions included: pregnant and lactating women; older individuals with risk of falling or fracture; dark skinned populations; and, obese persons.

She commented that while the recommendations were intended to adequately meet the physiological states and the needs of these special groups, there remains inadequate evidence of improved clinical outcomes with higher levels of circulating 25(OH)D in dark skinned persons or the obese. Emerging data and ongoing studies are expected to address vitamin D metabolism and physiological requirements in these populations.

Dr. Shapses considered the possibility that lower concentrations of 25(OH)D in Black Americans might explain the higher incidence of certain diseases (hypertension and cardiovascular disease) and mortality in this group when compared to White Americans. She focused on obesity as an example since the prevalence of obesity is higher in Black Americans and it is associated with multiple co-morbidities.

Dr. Shapses noted that serum 25(OH)D levels are low in obese individuals and inversely correlated with adiposity. It is generally assumed that adipose tissue acts as a depot for vitamin D. However, in obese individuals, if non-alcoholic fatty liver disease is also present, there may be altered vitamin D hydroxylation in the liver. Still, there is limited evidence for this latter hypothesis to explain low 25(OH)D in the obese.

In light of the higher incidence of obesity in Black populations (48%) compared to Whites (33%), Dr. Shapses presented data showing in obese individuals increased levels of PTH, estrogen, insulin, MCP-1, and other cytokines, and decreases in adiponectin, osteocalcin, 25(OH)D, ghrelin, and growth hormone. Nonetheless, the interesting paradox framing obesity states is that although obese individuals have normal BMD they show higher fracture risk.

Dr. Shapses then presented data that 25(OH)D levels raised with increasing weight loss (in healthy individuals) and that the increase was believed to be due to a release of 25(OH)D from adipose tissue. Not only was there a greater increase in 25(OH)D with more weight loss, but there was also a greater increase in 25(OH)D levels following vitamin D supplementation in those who lost more weight.

## Overview of discussions

The expert panel discussions focused on creating a better understanding of the factors affecting differences in vitamin D status and bone health in Black Americans versus White Americans. A secondary focus of the discussions was on the impact of vitamin D supplementation and the potential unintended consequences of current health care practices. The two distinct discussion sessions, each led by a moderator, are summarized below.

### Session 1

**What is the current state of the science on the vitamin D requirements for reducing risks of osteoporosis and fractures in adult Black vs. White Americans** *?*

*Moderator: Andy Hoofnagle, MD, PhD.*

Much of the discussion in this session focused on understanding the contribution (if any) that adiposity, genetics, and vitamin D supplementation might have on vitamin D status and bone health in Black vs. White Americans. With respect to obesity, the panelists agreed that there was clearly a strong association between obesity and low levels of 25(OH)D, and that 25(OH)D was likely sequestered in adipose tissue. However, as the panelists did not all believe that the greater prevalence of obesity in Black Americans vs. White Americans accounted for the racial differences in 25(OH)D, they did agree that obesity was likely a minor contributor to the overall paradox. It was mentioned that if an oversimplified view of the sequestering of vitamin D in adipose tissue was accepted, then at some point, a steady state should be reached and serum levels of 25(OH)D should normalize. As such, panelists acknowledged that additional factors that may impact the paradox and that warrant further exploration, include the existence of a potential cholesterol and lipid metabolism effect that’s been shown to be evident despite correcting for BMI.

To better understand the role of genetics with respect to the vitamin D paradox, the panelists discussed potential limitations of the GWAS approach. They reiterated the importance of the fact that GWAS only included White populations. As such, many variants in the genome remained unaccounted for. The CYP24A1 (24, 25 hydroxylase) polymorphism was discussed as one example. While variants at this locus showed a strong association with PTH levels in individuals enrolled in the GWAS, the association was shown to be of little use for explaining the high levels of PTH and/or PTH resistance in bone in Black Americans. In cases where loci variants from the GWAS were shown to replicate in Black Americans (e.g. polymorphisms associated with VDBP), there was an expressed concern with the lack of causality in the associations.

The panelists also pondered whether recommendations for vitamin D supplementation should differ according to life stage and/or across different subpopulations. There was a consensus that the IOM recommendations of 400-600 IU/day may be appropriate for various healthy subpopulations, but concerns were expressed about the misuse of threshold values for managing individuals’ health. Panelists agreed that the values should be used only as tools for assessing population trends. Of greater concern was the increasing rate of over-supplementation of vitamin D. One panelist commented, “We are a human experiment -- people take more vitamin D than ever before. Some athletic team coaches are supplementing to get players over 40 ng/mL.”

### Session 2

**Is the current approach for assessing vitamin D status (with respect to bone health) sufficient for all populations? What are potential unintended consequences of current assessments for vitamin D status on the health of Black Americans?**

*Moderator: Sue Shapses, PhD, RD.*

Panelists grappled with the appropriateness of current approaches being used to assess vitamin D status in all populations given the scarcity of available data on Black Americans and other subpopulations. It was suggested that 25(OH)D is likely not an ideal marker for measuring vitamin D status but rather is a good biomarker of exposure (sunlight and diet).

Despite these concerns, panelists ultimately agreed that, while not perfect, 25(OH)D is currently the best tool available for assessing vitamin D status. As such, the discussion focused on approaches for improving the use and/or applicability of current tools and recommendations. It was suggested that clarification is needed on whether assessment of vitamin D status should focus on determining risk of vitamin D deficiency or on determining risk of chronic disease. Clarification is also needed on the recommendations for safe upper limits in various subpopulations. Panelists were reminded that the Endocrine Society recommends 10,000 IU/day as an upper safe limit in the general population, while the IOM recommends 4000 IU/day as an upper safe limit in the general population. Yet, evidence was presented that risks of falls and fractures in Black Americans were shown to increase with doses as low as 2000 IU/day. Panelists believed that such lack of clarity was almost certain to result in negative unintended consequences in Black populations. Other suggestions for improved use of current tools and recommendations included focusing less on threshold values and more on IOM Recommended Daily Allowance (RDA) values and improving public health messaging regarding the potential for adverse effects following over-supplementation with high doses of vitamin D.

## Conclusion

The opinions, discussions, and data presented by the participants of the expert panel meeting illustrated that the vitamin D paradox in Black Americans is an important and relevant public health conundrum. Future discussions should include identifying crucial knowledge gaps impacting vitamin D that expand beyond the paradox, and that include conditions other than bone health. These initial discussions were intentionally focused only on the paradox since understanding the paradox, or factors that impact vitamin D levels and bone health in Black Americans, is the first step and may provide possible mechanisms for improving bone health in all populations. Various factors including adiposity, skin pigmentation, vitamin D binding protein polymorphisms, and genetics contributed to differences in 25(OH)D levels in Black vs. White Americans. However, there was no single factor that could fully explain the vitamin D paradox in Black Americans.

The panelists discussed both the lack of skeletal benefits from high doses of vitamin D supplementation in Black Americans as well as the skeletal risks of maintaining high serum levels of 25(OH)D in this population, noting that these levels need further refinement. In addition, the meeting highlighted the importance of addressing critical public health disparities to further advance the science of vitamin D. The lack of data on Black Americans as well as other subpopulations within the U.S. was of particular concern. An exploration of next steps will include opportunities (perhaps via a larger forum or workshop) for stimulating more research on vitamin D with respect to bone health, implications beyond bone health, and its impact on various segments of the population.
